# Mechanism of Core Browning in Different Maturity Stages of ‘Yali’ Pears During Slow-Cooling Storage and PbRAV-Mediated Regulation

**DOI:** 10.3390/foods14122132

**Published:** 2025-06-18

**Authors:** Bing Deng, Qingxiu Li, Liya Liang, Hongyan Zhang, Xiaoyu Zhang

**Affiliations:** 1 College of Food Science and Engineering, Shanxi Agricultural University, Taigu 030801, China; 2 College of Food Science and Biological Engineering, Tianjin Agricultural University, Tianjin 300392, China

**Keywords:** ‘Yali’ pear, core browning, transcriptome analysis, PbRAV transcription factors

## Abstract

This study investigated the impact of slow cooling on browning and fruit quality at three maturity stages (early, mid and late). Slow cooling reduced core browning in early/mid-harvest pears, as the browning indexes of early-, middle- and late-harvested ‘Yali’ pears at 60 d were 0.13, 0 and 0.1, respectively, preserving firmness and soluble solids. Transcriptomic analysis revealed that upregulated genes in ‘Yali’ pears facilitated stress adaptation via enhanced catalytic activity and phosphorylation. Mid-harvested pears exhibited activation of phosphorus metabolism and DNA repair mechanisms to maintain cellular homeostasis, whereas the late-harvested counterparts showed significant suppression of photosynthesis-related pathways and pyrimidine metabolism, which collectively accelerated senescence progression. Universal downregulation of hormone-response pathways such as ethylene and auxin revealed systemic stress adaptation decline. Then, the PbRAV transcription factors’ role was also studied. EMSA confirmed that GST-PbRAV2 binds to the PbLAC15 promoter, linking RAV2 to laccase regulation. Overripe pears showed PbRAV2 dysregulation, impairing LAC15 suppression and accelerating browning. Findings provide a theoretical basis for using slow cooling to mitigate browning in pear storage.

## 1. Introduction

The ‘Yali’ pear (*Pyrus bretschneideri* Rehd.), a prominent traditional cultivar originating from Hebei province, China, is highly valued for its distinctive fragrance and delicate aroma [1,2]. However, core browning frequently manifests during postharvest storage, as a notable physiological affliction in pears, significantly diminishing commodity value and impacting both export volume and revenue, remaining undetectable via visual inspection. Prior research has established a definitive correlation between the manifestation of core browning and the onset of fruit senescence. The progression of senescence is significantly accelerated by a decline in ethylene biosynthesis during extended storage durations [3]. The progression of core browning in ‘Yali’ pears can be succinctly characterized as follows: Initially, the ‘Yali’ pear core shows no browning. With prolonged storage, browning begins in the ovary, progressing to complete core browning and extending into the flesh. Browning consistently initiates in the core and spreads outward into the mesocarp [4].

The ‘Yali’ pear is prone to core browning during postharvest storage, a significant postharvest physiological disorder [5]. This browning phenomenon detrimentally affects the quality attributes of ‘Yali’ pears, diminishing their palatability and, consequently, impacting marketability. In ‘Yali’ pears, browning is primarily associated with the modulation of specific genes and associated enzymatic activities. Phenolic compounds serve as the principal substrates in this process. Under aerobic conditions, these phenolic substrates are oxidized by polyphenol oxidase, yielding quinones, which subsequently accelerate browning [6]. Core browning in ‘Yali’ pears is influenced by a multitude of factors, including harvest maturity, fruit quality, storage temperature, CO_2_ levels, storage protocols, and exogenous preservative applications. These variables modulate the fruit’s sensitivity to temperature fluctuations [4]. At present, low-temperature storage is the most commonly used storage method in the preservation of fruits and vegetables. Low-temperature storage can delay energy consumption and reduce their metabolic rate, thus inhibiting the reproduction of bacteria and microorganisms, delaying senescence, effectively prolonging the storage period and slowing down the occurrence of browning [7,8]. For the ‘Yali’ pear, appropriate maturity, combined with different cooling treatment during low-temperature storage, can effectively inhibit the occurrence of core browning [7,9]. For example, postharvest ‘Yali’ pears at advanced maturity treated with rapid cooling show that it effectively suppresses ethylene biosynthesis and respiratory activity. This physiological modulation subsequently retards the senescence process, thereby preserving fruit quality attributes [4]. Furthermore, this effect is correlated with elevated activities of superoxide dismutase (SOD), catalase (CAT), peroxidase (POD), and glutathione reductase (GR), alongside diminished lipoxygenase (LOX) activity. Furthermore, the slow-cooling protocol delayed membrane lipid peroxidation, suppressed malondialdehyde (MDA) accumulation, reduced levels of $O_{2}^{-}$ and H_2_O_2_, and attenuated cell membrane damage [7].

Initial investigations have elucidated the correlation between membrane lipid peroxidation, ethylene metabolism, and the incidence of core browning in ‘Yali’ pears; however, the underlying molecular mechanisms remain obscure. Within the plant system, transcription factors (TFs) modulate the transcriptional activity of structural genes through interaction with cognate cis-regulatory elements located within their promoter regions [10]. Transcription factors (TFs) belonging to the WRKY, bZIP, NAC, and MYB families have been frequently implicated in the regulation of fruit browning [11,12,13,14]. This study utilized ‘Yali’ pears from Xinji City, Hebei Province, as experimental material. ‘Yali’ pears were subjected to slow-cooling treatments at three maturity stages (early, mid, and late), determined based on the full bloom and harvest periods to investigate their effects on core browning and physiological quality during storage. Transcriptome analysis of the fruit core was then performed to identify key transcription factors regulating browning, and to systematically characterize the transcriptional regulatory network of RAV transcription factors in the core browning pathogenesis. The aim was to elucidate the molecular mechanisms of cooling-mediated browning inhibition, providing a theoretical basis for optimizing postharvest preservation strategies.

## 2. Materials and Methods

### 2.1. Plant Material and Postharvest Treatment

‘Yali’ pears sourced from Xinji City, Hebei Province, served as the experimental material. Fruits were harvested 145 days after bloom (early-maturity), 155 days after bloom (mid-maturity), and 165 days after bloom (late-maturity). Uniformly sized fruits (about 90 mm), devoid of pest infestation and mechanical injury, were selected. For each maturity stage, 3 replicates, each comprising approximately 80 fruits, were utilized. Following harvest, the fruits were immediately transported to the cold storage facility at Tianjin Agricultural College. Pre-cooling was initiated for 24 h, succeeded by a controlled cooling regime. The slow-cooling protocol comprised pre-cooling at 12 °C for 24 h, followed by a progressive decrease to 0 ± 0.5 °C over 30 d, with a 2 °C reduction every 5 d. Relative humidity was sustained at 80–85%. Core browning and the physiological quality of ‘Yali’ pears were assessed at 30 d intervals for each maturity group. Simultaneously, core tissue samples were excised and immediately subjected to flash-freezing in liquid nitrogen. These samples were subsequently stored at −80 °C until further analysis.

### 2.2. Determination of Core Browning and Physiological Quality of ‘Yali’ Pears After Slow-Cooling Treatment

The core browning index and physiological quality of ‘Yali’ pears was assessed according to the methodology described by Li et al. [7] and Zhang et al. [4], with some modification.

#### 2.2.1. Determination of Core Browning Index of ‘Yali’ Pear

A total of 30 pears were randomly selected at each maturity stage. These were subsequently divided into three replicates, each comprising 10 pears. Core browning in ‘Yali’ pears was monitored and documented at intervals of 0, 30, 60, 90, 120, 150, 180, and 210 d of storage, and the core browning index was computed as detailed below:

$$core\;browning\;index=\frac{\sum \left(Browning\;grade\times The\;number\;of\;fruits\;of\;this\;grade\right)}{The\;highest\;browning\;grade\times Check\;the\;number\;of\;fruits}$$

#### 2.2.2. Determination of Soluble Solids (SSC) Content

A total of 15 fruits were randomly sampled from each treatment group, with every 5 fruits forming one group, and 3 replications were performed. After peeling 5 fruits with a knife, the pulp portion was cut into small pieces, mixed and juiced, and a saccharimeter was used to determine the soluble solids content of the ‘Yali’ pear.

#### 2.2.3. Firmness Determination

A total of 15 fruits were randomly selected for the experiment. The firmness of ‘Yali’ pear fruits was assessed utilizing a TA-XT plus texture analyzer, with measurements expressed in kg cm^−2^.

#### 2.2.4. Determination of Respiratory Intensity

A total of 12 ‘Yali’ pears were randomly selected and apportioned into three treatment groups. These were designated and reserved for subsequent analyses. Subsequently, the grouped ‘Yali’ pears were housed within modified Lekou boxes and sealed for 1 h. Gas samples were extracted from each box using a 1 mL medical syringe, with four needles per box (three for replication and one backup). The respiratory intensity of the ‘Yali’ pears was assessed using a respiration detector. Determination of respiratory intensity was performed using a CA-10 CO_2_ analyzer and calculated according to the formula in Zhang et al. [4].

#### 2.2.5. Determination of Ethylene Release

The gas was collected in accordance with the procedure used for measuring respiratory intensity, and the samples obtained were determined using a Shimadzu GC-14 gas chromatograph. The experimental parameters were set as follows: the GDX-502 stainless-steel-packed column was the chromatographic column; the detector was a hydrogen-flame ionization detector, and the carrier gas was N2; the temperature of the inlet was 60 °C, the temperature of the column temperature box was 60 °C, and the temperature of the detector was 60 °C. The calculation formula is shown below, and the unit is expressed in μL kg^−1^·h^−1^ µL.

$$Ethylene\;release=\frac{C\times V}{M\times T\times 1000}$$

in which C: ethylene content in the sample gas with unit of µL L^−1^;
V: volume of the enclosed space, unit is mL;
M: the mass of fruits and vegetables, the unit is kg;
T: smothering time; the unit is h.

#### 2.2.6. Determination of Weight Loss

We measured the mass of fixed ‘Yali’ pears every 30 d with an electronic balance and then calculated the change in fruit weight. The calculation formula was as follows:

$$Weight\;loss\;rate\;(\%)=\frac{weight\;of\;the\;initial\;sample-weight\;of\;the\;current\;sample}{weight\;of\;the\;initial\;sample}\times 100$$

### 2.3. Transcriptome Sequencing

The samples fruit core samples were frozen in liquid nitrogen stored in an ultra-low-temperature refrigerator at −80 °C. The frozen samples were sent to Beijing Novozymes for transcriptome sequencing.

### 2.4. Analysis of Cis-Acting Elements

The analysis of cis-acting elements in *Pyrus bretschneideri* RAV (PbRAV) was performed in PlantCARE (https://bioinformatics.psb.ugent.be/webtools/plantcare/html/) accessed on 8 September 2022.

### 2.5. RNA Isolation and cDNA Synthesis

The improved CTAB method was used to extract total RNA from each sampling point of ‘Yali’ pear fruit. The quality of RNA extraction was detected by 1% agarose gel electrophoresis, the concentration and purity of RNA were detected using a NanoDrop 2000 spectrophotometer (Thermo, Waltham, MA, USA). Approximately 2 μL RNA was used to produce cDNA with an HiScript^®^Ⅱ 1st Strand cDNA synthesis kit (R212, vazyme, Nanjing, China).

### 2.6. Quantitative Real-Time PCR (RT-qPCR)

In RT-qPCR analyses, cDNA was diluted with ddH_2_O at a ratio of 1:10. Gene-specific primers were designed using Primer 5.0 software (Table S1). RT-qPCR was performed on the QuantageneQ225 Real-time PCR system (Q225, Novegene, Beijing, China) with ChamQ SYBR Color qPCR Master Mix (Q421, vazyme, Nanjing, China). The RT-qPCR analyses were performed with three replicates to ensure accurate results. The *Pyrus bretschneideri* actin (GU830958.1) was used as reference gene, and the relation gene expression level of each gene was calculated using the 2^−ΔΔCT^ method.

### 2.7. Identification of Downstream Target Genes of PbRAV Across the Entire Genome

The binding sites of the PbRAV transcription factor were predicted using the JASPAR online database. Subsequently, the complete *Pyrus bretschneideri* genome data was downloaded from the NCBI database. The upstream 2000 bp promoter sequences of all protein-coding genes in *P. bretschneideri* were extracted using TBtoolsV1.098 software. Finally, Kyoto Encyclopedia of Genes and Genomes (KEGG) pathway enrichment analysis was performed on the putative downstream target genes of the PbRAV transcription factor using the OmicShare tools [15]. Gene Ontology (GO) enrichment analysis was performed using the R package clusterProfiler (v4.6.2). The hypergeometric test was applied with all protein-coding genes detected in RNA-seq as the background gene set. Terms with FDR-adjusted *p*-values < 0.05 were defined as significantly enriched.

### 2.8. Protein Expression and Purification and Electrophoretic Mobility Shift Assays (EMSA)

The methodology adhered to the protocols described by Fan et al. [16] and Tan et al. [17], alongside the operational guidelines provided by the Beyotime EMSA Chemiluminescence Kit (GS009).

### 2.9. Statistical Analysis

Statistical analysis was performed using Excel 2016. Origin was used for drawing. Values are represented as the mean of three independent biological replicates. IBM 22 (IBM Inc., Armonk, NY, USA) statistics software was used for the variance analysis of data, and *p* ≤ 0.05 was considered significant.

## 3. Results

### 3.1. Effects of Slow-Cooling Treatment on Core Browning Index and Physiological Indicators of Different Maturity ‘Yali’ Pear

**Figure 1:**
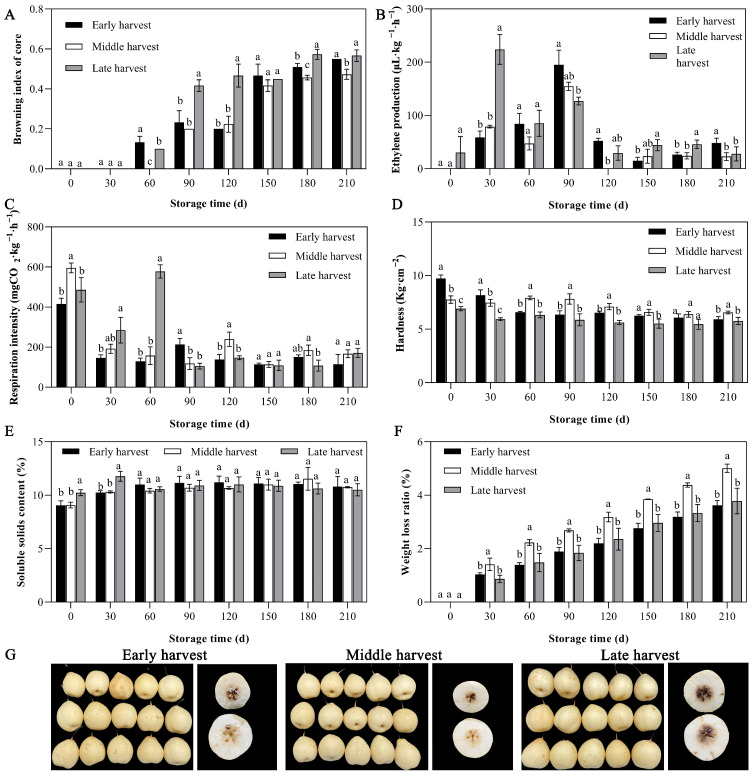
Effect of slow-cooling treatment on core browning index (**A**), ethylene production (**B**), respiration intensity (**C**), hardness (**D**), soluble solids (**E**), the weight loss rate (**F**) of ‘Yali’ pears at different maturity stages, and the core browning of ‘Yali’ pears at different maturity stages at 210 d of storage (**G**). Variations in lowercase letters indicate a statistically significant difference (*p* < 0.05) among five groups simultaneously. Y-bars represent SD.

The browning index of ‘Yali’ pears at different maturity stages gradually increased with prolonged storage time (Figure 1A). During the early storage period (0–30 d), there was no core browning in the ‘Yali’ pears. Early- and late-harvested ‘Yali’ pears first began to show browning after 60 d of storage, and the browning indexes were 0.13 and 0.1, respectively. The browning index of middle-harvested ‘Yali’ pears was 0.2 when it first appeared, at 90 d of storage. At the end of storage (180–210 d), it was found that the core browning index of different maturity ‘Yali’ pear was as follows: late-harvest > early-harvest > middle-harvest. The results showed that the ‘Yali’ pears with either higher or lower harvest maturity were not suitable for slow-cooling treatment.

The overall trend in ethylene release in fruits first rose to a peak and then decreased (Figure 1B). The ethylene peak timing varied with fruit maturity: late-harvested ‘Yali’ pears exhibited the first peak at 30 days (223.95 μL·kg^−1^·h^−1^), while at 90 days both early- and middle-harvested fruits showed peaks at 195.02 and 154.64 μL·kg^−1^·h^−1^, respectively. From 180 to 210 days, ethylene release increased, correlating with accelerated senescence and browning, as reflected in the browning index. Throughout storage, late-harvested pears consistently released more ethylene, indicating that higher maturity adversely affects long-term preservation post-slow cooling.

During the storage process of ‘Yali’ pear, the respiratory intensity of ‘Yali’ pears harvested at different maturity stages changed as shown in Figure 1C. Overall, the trend showed an initial decline, then a rise, and finally a decline again. At 60 d of storage, the respiratory peak of late-harvested ‘Yali’ pears first appeared, reaching 577.63 mg CO_2_ kg^−1^·h^−1^; at 90 d of storage, the respiratory peak of early-harvested ‘Yali’ pears occurred, reaching 213.9 mg CO_2_ kg^−1^·h^−1^; at 120 d, the respiratory peak of middle-harvested ‘Yali’ pears appeared, reaching 239.47 mg CO_2_ kg^−1^·h^−1^. During the whole storage period, the late-harvested ‘Yali’ pears were the first to exhibit the respiratory peak, indicating that the fruit with higher maturity was not suitable for long-term preservation under the slow-cooling treatment. Based on the timing of the respiratory peak and the sudden increase in the core browning index in ‘Yali’ pear, the earlier the respiratory peak occurred, the more prone ‘Yali’ pear was to core browning.

Hardness is a key indicator of fruit quality. The hardness of ‘Yali’ pear showed a decreasing trend during the whole storage period. In 0 d of storage, the hardness of ‘Yali’ pear was 9.72, 7.76, and 6.90 kg cm^−2^ in the early, middle, and late harvesting stages, respectively (Figure 1D). At 30 d of storage, the hardness of ‘Yali’ pear was as follows: early harvesting > middle harvesting > late harvesting, which indicated that the higher the maturity, the lower the initial hardness of the fruits. With the prolongation of storage time, the fruit gradually senesced. At the end of storage (180–210 d), the firmness of ‘Yali’ pear fruit was in the following order: middle harvesting > early harvesting > late harvesting, indicating that the hardness of the middle-harvested ‘Yali’ pear fruit declined the slowest after slow-cooling treatment, and that it can better maintain the fruit quality of ‘Yali’ pear.

With the prolongation of storage time, the soluble solids content (SSC) of ‘Yali’ pear fruits of three maturity levels showed a trend of first increasing and then decreasing (Figure 1E). At 0 d of storage, the SSC content of early-, middle-, and late-harvested ‘Yali’ pears was 9.05%, 9.06%, and 10.23%, respectively. The SSC content of late-harvested ‘Yali’ pears was higher in the pre-storage period, and in the late storage period (180–210 d), the soluble solids content of late-harvested ‘Yali’ pears was slightly lower than that of the early-harvested and middle-harvested pears.

The weight loss of ‘Yali’ pears exhibited a progressive increase with prolonged storage duration (Figure 1F). Within the initial 30 days of storage, the weight loss, contingent upon the maturity stage at harvest, followed this order: fruit harvested at the middle stage > early stage > late stage (early stage: 1.03%; middle stage: 1.41%; late stage: 0.86%). Over the entire storage period (180–210 days), the weight loss rate of the fruit was ordered as follows: middle stage > late stage > early stage.

### 3.2. Expression Trend Analysis of Total DEGs

**Figure 2:**
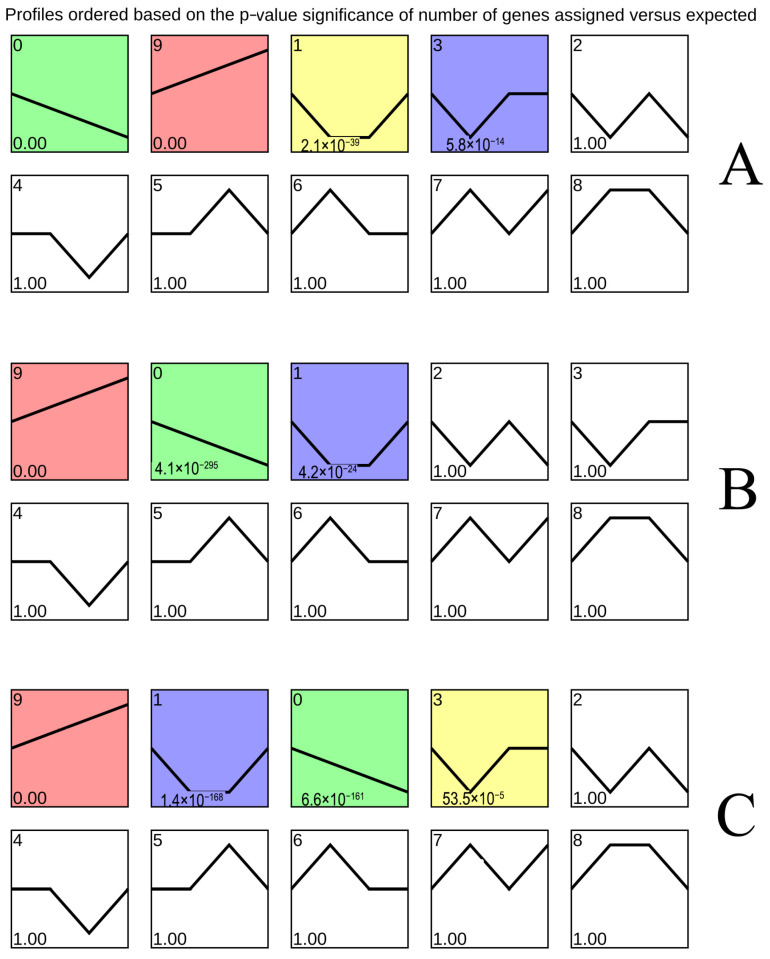
Impact of slow cooling on DEG expression trends in ‘Yali’ pears harvested at different maturity stages. DEG dynamics during storage (0, 30, 120, and 210 days) in early-harvested (**A**), middle-harvested (**B**), and late-harvested (**C**) ‘Yali’ pears.

Figure 2 depicted differential gene expression (|log_2_FC| > 0, padj ≤ 0.05) in ‘Yali’ pears during early, middle, and late maturity. Module 9 shows upregulation, with DEG counts of 6644, 7209, and 8382, respectively, increasing with storage time. Conversely, module 0 exhibits downregulation, with 6169, 5951, and 5460 DEGs, respectively, decreasing over time.

### 3.3. GO Enrichment Analysis of Different Expression Trend Modules

**Figure 3:**
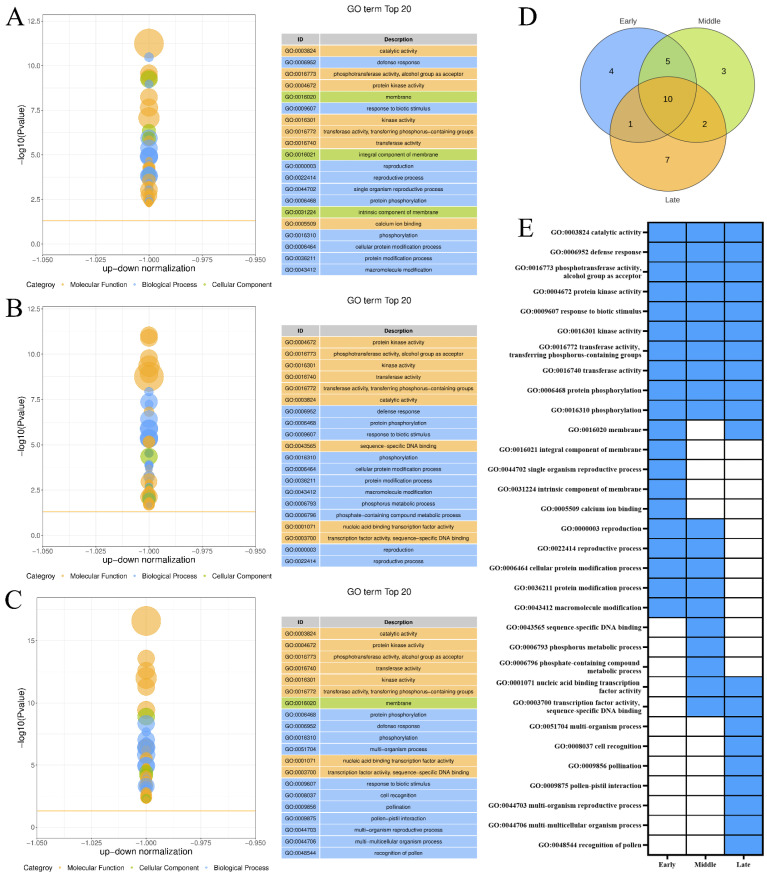
GO enrichment of upregulated genes (Profile 9) in ‘Yali’ pears at different maturities after slow-cooling treatment. (**A**–**C**) GO terms for early-, middle-, and late-maturity samples. (**D**) Venn diagram showing shared and unique enriched GO terms among the three maturity stages. (**E**) Heatmap displaying enrichment status: blue indicates enriched, while white indicates not enriched.

The top 20 GO terms enriched by the DEGs included in the upregulated expression trend Profile 9 of the three maturity levels of ‘Yali’ pears after the slow-cooling treatment (Figure 3A–C). Subsequently, the logical linkage analysis of the top 20 GO terms for the three maturity levels was carried out (Figure 3D,E), and three maturity stages of ‘Yali’ pears were found to be commonly enriched in 10 GO terms, where the catalytic activity is closely related to core browning in ‘Yali’ pears. Between maturity stages, one GO term was shared between early and late-harvested pears, while mid-harvested pears shared four terms with early and two terms with late-harvested fruits. Compared to the other two maturity stages, the mid-harvested ‘Yali’ pears were primarily enriched in GO terms, such as sequence-specific DNA binding, phosphorus metabolic process, and phosphate-containing compound metabolic process. This may be one of the reasons why the mid-harvested ‘Yali’ pears exhibited a lower degree of core browning under slow-cooling treatment.

**Figure 4:**
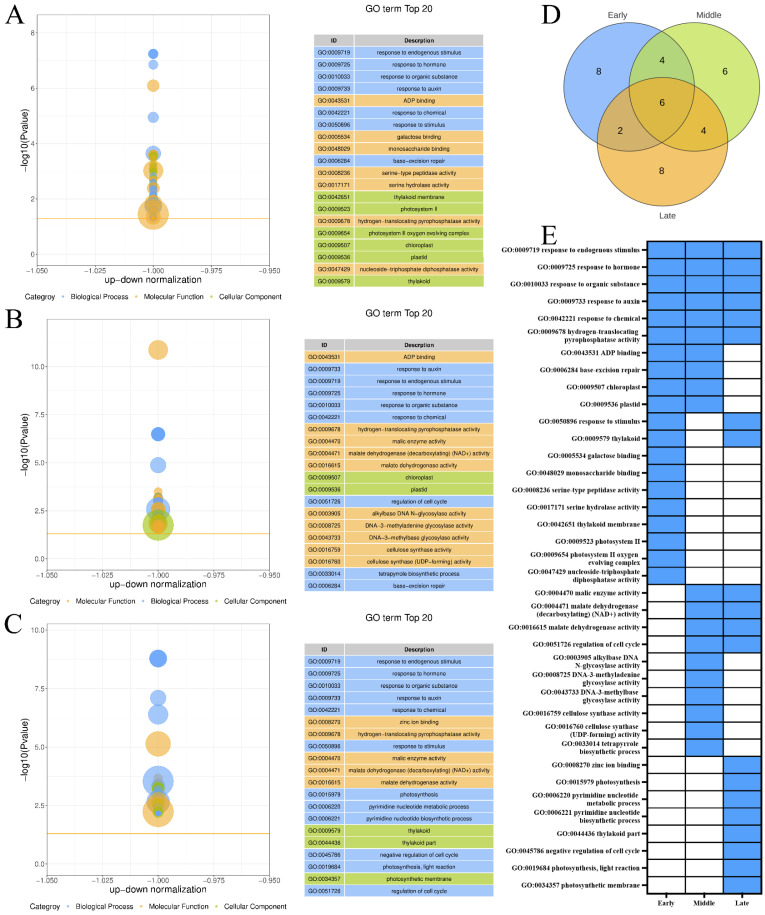
GO enrichment of downregulated genes (Profile 0) in ‘Yali’ pears at different maturity stages after slow-cooling treatment. (**A**–**C**) GO terms for early-, middle-, and late-maturity samples. (**D**) Venn diagram showing shared and unique enriched GO terms among the three maturity stages. (**E**) Heatmap displaying enrichment status: blue indicates enriched, while white indicates not enriched.

The top 20 GO terms enriched by the DEGs included in the downregulated expression trend Profile 0 across the three maturity levels of ‘Yali’ pears after the slow-cooling treatment (Figure 4A–C). Figure 4D shows that the three maturity stages of ‘Yali’ pears were co-enriched to six GO terms, and eight, six, and eight GO terms were enriched, respectively. The late-harvested ‘Yali’ pears exhibited significant enrichment in terms related to zinc ion binding, photosynthesis, pyrimidine nucleotide metabolic process, pyrimidine nucleotide biosynthetic process, thylakoid part, negative regulation of the cell cycle, photosynthesis, light reaction, and photosynthetic membrane (Figure 4E). This suggested that these processes are inhibited in late-harvested ‘Yali’ pears, as storage time increases. This enrichment may contribute to the increased core browning observed in late-harvested ‘Yali’ pears subjected to slow-cooling treatment.

### 3.4. KEGG Enrichment Analysis of Different Expression Trend Modules

**Figure 5:**
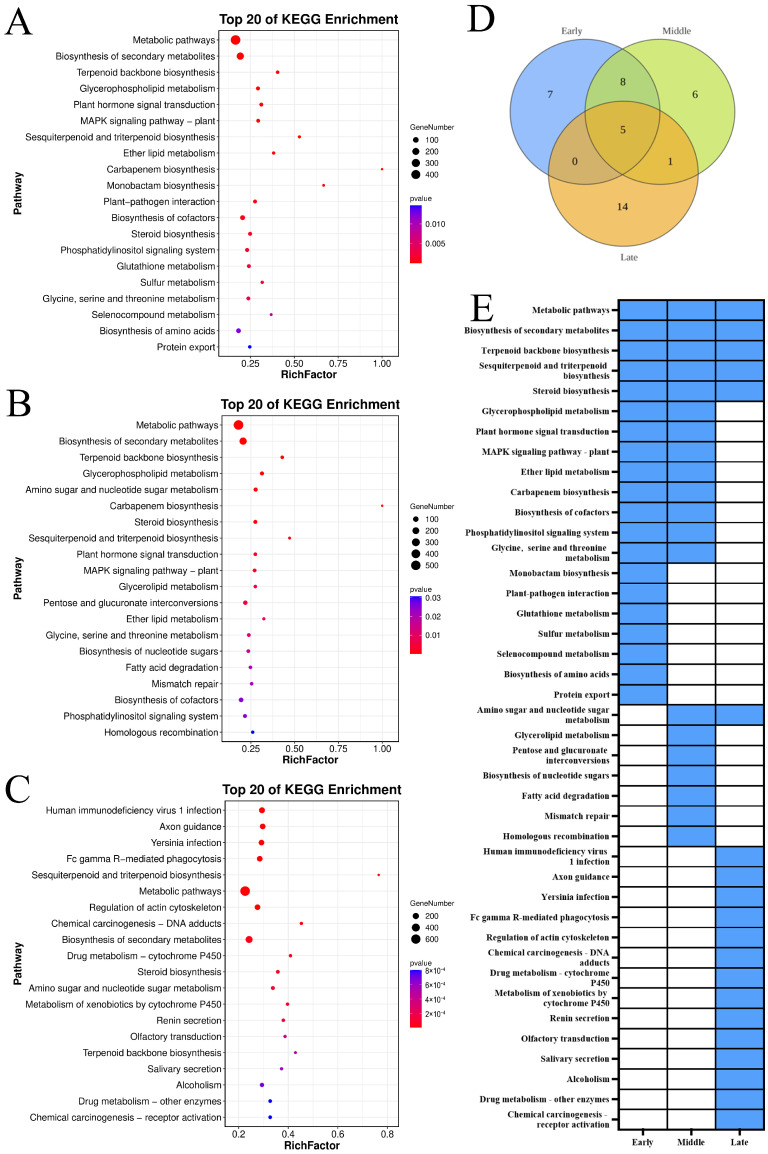
KEGG enrichment of upregulated genes (Profile 9) in ‘Yali’ pears at different maturity stages after slow-cooling treatment. (**A**–**C**) KEGG pathway for early-, middle-, and late-maturity samples. (**D**) Venn diagram showing shared and unique enriched KEGG pathways among the three maturity stages. (**E**) Heatmap displaying enrichment status: blue indicates enriched, while white indicates not enriched.

The KEGG pathway analysis depicted in Figure 5 indicated that the genes upregulated during the slow-cooling treatment across three ‘Yali’ pear maturity stages are significantly enriched in metabolic pathways, secondary metabolite biosynthesis, terpenoid backbone biosynthesis, sesquiterpenoid and triterpenoid biosynthesis, and steroid biosynthesis. Early- and mid-harvested pears share enrichment in eight KEGG pathways, including glycerophospholipid metabolism and plant hormone signal transduction, suggesting delayed core browning. Mid- and late-harvested pears share enrichment in amino sugar and nucleotide sugar metabolism. Additionally, mid-harvested pears are predominantly enriched in glycerolipid metabolism, pentose and glucuronate interconversions, nucleotide sugar biosynthesis, fatty acid degradation, mismatch repair, and homologous recombination pathways, contributing to delayed core browning onset.

**Figure 6:**
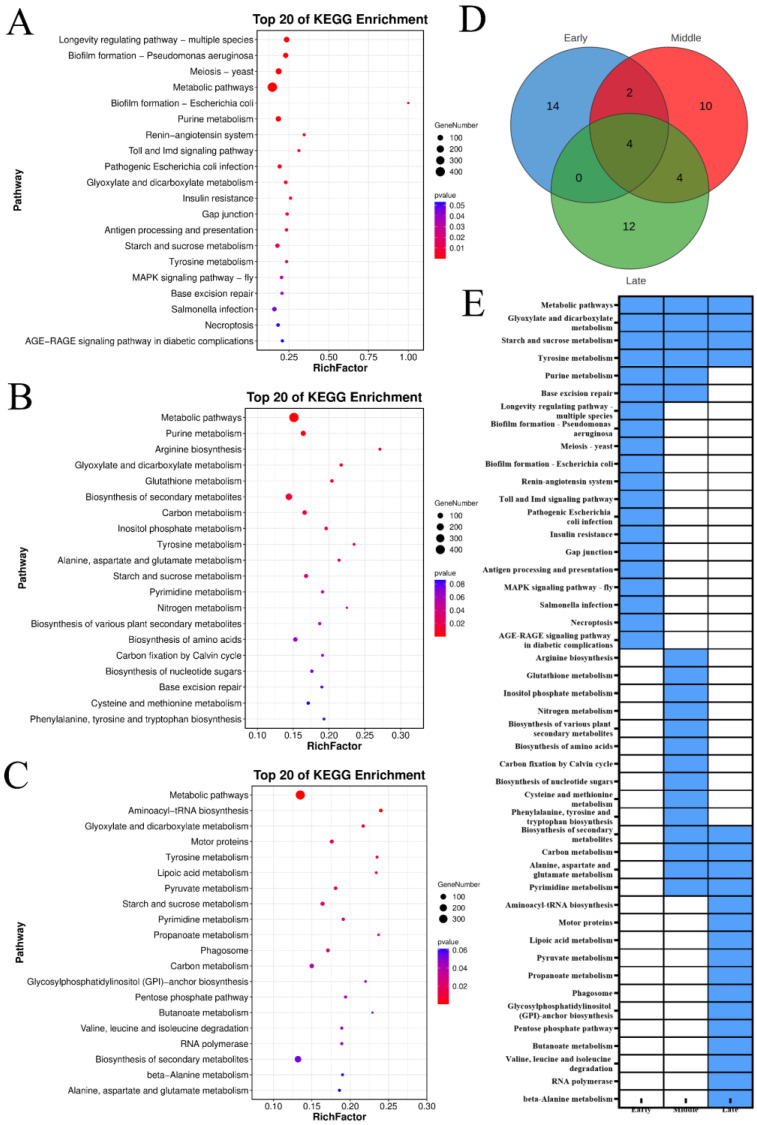
KEGG enrichment of downregulated genes (Profile 0) in ‘Yali’ pears at different maturity stages after slow-cooling treatment. (**A**–**C**) KEGG pathway for early-, middle-, and late-maturity samples. (**D**) Venn diagram showing shared and unique enriched KEGG pathways among the three maturity stages. (**E**) Heatmap displaying enrichment status: blue indicates enriched, while white indicates not enriched.

The KEGG enrichment analysis indicated that the downregulated genes across three maturity stages of ‘Yali’ pears post-slow cooling were predominantly enriched in metabolic pathways, glyoxylate and dicarboxylate metabolism, starch and sucrose metabolism, and tyrosine metabolism (Figure 6). Additionally, the genes downregulated in early- and mid-harvested pears were commonly enriched in purine metabolism and base excision repair pathways. The downregulated genes in mid- and late-harvested pears were enriched in four KEGG pathways, while the upregulated genes across early, mid-, and late-harvested stages were enriched in 14, 10, and 12 pathways, respectively. Late-harvested pears showed significant enrichment in pyruvate metabolism, GPI-anchor biosynthesis, pentose phosphate pathway, butanoate metabolism, and amino acid degradation pathways, indicating that suppression of these pathways may underlie core browning in ‘Yali’ pears.

### 3.5. Analysis of Expression Patterns and Regulatory Mechanisms of PbRAV in Core Browning of ‘Yali’ Pear

#### 3.5.1. Identification of PbRAV Transcription Factors

Multiple RAV transcription factors were identified as key regulators in the core browning of ‘Yali’ pear, with four genes (*RAV1-4*) showing differential expression. A cluster analysis revealed stage-specific expression patterns during storage; *RAV1* and *RAV2* were upregulated at mid-harvest maturity, while *RAV4* was downregulated at this stage (Figure S1).

#### 3.5.2. Analysis of the PbRAV Gene Promoter Sequence

Table S2 lists the identified cis-acting elements associated with growth, development, abiotic stress, and hormonal regulation, such as CAT-box, TC-rich, LTR, MBS, ABRE, GARE-motif, P-box, TATC-box, CGTCA-motif, and AuxRR, indicating PbRAV’s potential role in hormone metabolism and stress response modulation.

#### 3.5.3. Relative Expression of PbRAV Genes in the Core of ‘Yali’ Pears at Varying Maturity Stages Following Slow-Cooling Treatment

**Figure 7:**
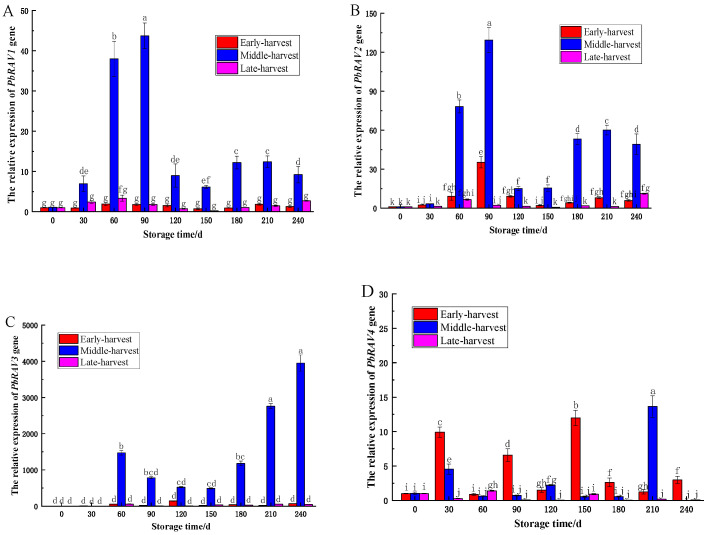
Relative expression of *PbRAV* genes in the core of ‘Yali’ pears at different maturity stages following slow-cooling treatment (Note: (**A**): *PbRAV1*; (**B**): *PbRAV2*; (**C**): *PbRAV3*; (**D**): *PbRAV4*). Variations in lowercase letters indicate a statistically significant difference (*p* < 0.05) among the five groups.

The relative expression of *PbRAV1* and *PbRAV2* genes in the ‘Yali’ pear core exhibited an initial increase, followed by a decrease, then a subsequent rise during storage (Figure 7). Specifically, both genes showed rapid upregulation early in storage, declining at 120 days, then increasing again from 180 to 240 days. The increase in *PbRAV2* expression was significantly greater than that of PbRAV1 throughout. In mid-harvested fruit, both genes peaked at 90 days (*p* < 0.05), with lower levels in early and late harvests. *PbRAV1* was downregulated at 30, 150, and 180 days in early-harvested fruit, and at 120 and 150 days in late-harvested fruit; *PbRAV2* was downregulated at 150 days in late-harvested fruit.

This study indicated that *PbRAV3* was generally upregulated during storage in ‘Yali’ pears across all harvest stages, except at 30 days in the mid-harvested fruit, with peak expression at 240 days (*p* < 0.05). Conversely, *PbRAV4* was downregulated at multiple time points in early-harvested fruit and at 60 days in mid-harvested fruit, with the highest expression at 210 days (13.66) in mid-harvested samples. In the late-harvested pears, *PbRAV4* was upregulated at 60 days and downregulated thereafter. These expression patterns correlated with core browning indices (late-harvested > early-harvested > mid-harvested), suggesting that *PbRAV* gene upregulation may inhibit core browning in mid-harvested fruit, while *PbRAV4* downregulation may promote core browning in late-harvested pears.

#### 3.5.4. Prediction and Analysis of Downstream Target Genes of PbRAV Based on the Complete Genome

Binding sites prediction revealed that PbRAV can specifically bind to DNA sequences with the motifs 5′-CAACA-3′ and 5′-CACCTG-3′. A cis-element analysis of the promoter sequences of all protein-coding genes in the genomic data identified a potential presence of 140,798 CAACA binding sites and 10,980 CACCTG binding sites for the PbRAV transcription factor. This suggested a substantial number of potential downstream target genes for the PbRAV transcription factor within the *Pyrus bretschneideri* genome. A KEGG enrichment analysis was performed on the potential downstream target genes of the PbRAV transcription factor. The results indicated that the PbRAV transcription factor may regulate the synthesis and accumulation of metabolites, lipid metabolism, and signal transduction pathways. Laccase is one of the potential downstream target genes of the PbRAV transcription factor (Figure S2). This study selected the LAC15 gene (containing two LTR elements) to initiate an investigation into the regulation of target genes by the PbRAV2 transcription factor.

#### 3.5.5. Validating the Binding of the PbRAV2 Transcription Factor with the PbLAC15 Gene Promoter

**Figure 8:**
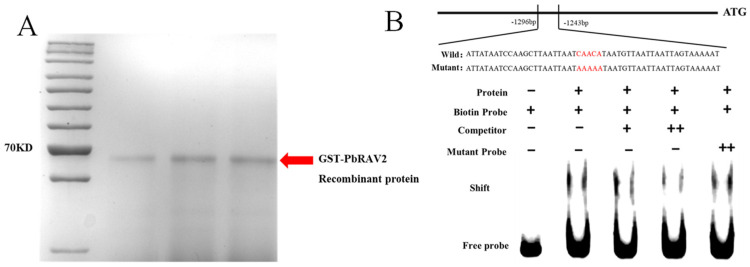
Purification of GST-PbRAV2 protein (**A**) and EMSA verifies that PbRAV2 binds to the LAC15 promoter (**B**).

The purified GST-PbRAV2 protein displayed a band size matching the predicted molecular weight, confirming its suitability for EMSA. The EMSA results showed a single probe band without protein, which shifted upon GST-PbRAV2 addition, indicating binding. The binding was competitively inhibited by excess unlabeled probe but was unaffected by mutated probe, demonstrating specific interaction with the PbLAC15 promoter (Figure 8).

## 4. Discussion

During extended cold storage of ‘Yali’ pears (up to 1 year), the fruit is prone to core browning. Controlling the cooling rate of postharvest storage of ‘Yali’ pear can effectively inhibit the occurrence of core browning, and the most significant effect of inhibiting core browning of postharvest ‘Yali’ pear is slow cooling combined with low-temperature storage [7]. To explore which maturity stage was more appropriate for storage under slow-cooling treatment, ‘Yali’ pears at early, middle, and late maturity stages were harvested and stored under slow-cooling treatment, then their physiological indexes were measured during storage, and the changes in physiological metabolism were observed. It was found that the appropriate maturity stage of ‘Yali’ pear combined with slow-cooling treatment could reduce the browning phenomenon. Early-harvested ‘Yali’ pears exhibited browning at 60 days post-storage, while middle-harvested pears browned at 90 days. Core browning was most severe in late-harvested pears, consistent with the findings of Yan [18]. Slow cooling is unsuitable for all mature ‘Yali’ pears; lower internal browning incidence in middle-harvested fruits [19] suggests that cooling rate and fruit maturity should be optimized to mitigate core browning.

Core browning correlates with fruit senescence onset, influenced by declining ethylene synthesis during storage [3]. ‘Yali’ pears exhibit a peak in ethylene production, with timing varying by harvest maturity—earliest in late-harvested, delayed in early- and mid-harvested. A secondary increase in ethylene production during 180–210 days of storage accelerates senescence and core browning. Elevated ethylene in late-harvested pears indicates reduced storability under slow cooling. Respiratory activity shows an initial decline, then an increase, then a decline, with earlier peaks in late-harvested pears. The timing of respiratory peaks aligns with browning onset, highlighting that earlier respiratory surges and higher maturity impair long-term storage. Flesh browning disorder correlates with nutrient composition in mesocarp tissues [20]. Firmness declines progressively during storage, inversely related to harvest maturity; mid-stage harvest retains firmness better, indicating delayed senescence. Rising demand for high-quality fruit emphasizes titratable acidity (TA) and soluble solids content (SSC) as quality indicators [5,21]. The SSC initially increases and then decreases, with late-harvested pears showing higher early SSC but lower later values compared to earlier harvests. Weight loss accumulates over time, peaking in mid-stage harvest, reflecting maturity-dependent water permeability. Results suggest late harvest accelerates ethylene-induced senescence and browning, while mid-stage harvest balances firmness retention and physiological activity, favoring extended storage under slow cooling.

The RNA-Seq analysis of ‘Yali’ pears at various maturity stages revealed conserved GO terms—catalytic activity, defense response, and phosphorylation—among upregulated DEGs, indicating shared stress response and signaling pathway activation during core browning under slow cooling. The correlation between mineral nutrient concentrations and the incidence of physiological disorders in postharvest fruit is well established. Phosphorus (P), a key component of energy metabolism, has been implicated in the development of internal browning in ‘Rocha’ and ‘Conference’ pears, and the results of Wang indicated that the P content was lower in the browning tissue compared to the healthy fruit [20]. Notably, mid-harvested pears exhibited unique enrichment in phosphorus metabolism and DNA-binding processes (e.g., sequence-specific DNA binding), potentially enhancing phosphorus metabolism, which may contribute to their reduced core browning susceptibility. Conversely, downregulated DEGs revealed distinct maturity-specific suppression patterns. Late-harvested pears demonstrated significant inhibition of photosynthesis-related pathways (e.g., thylakoid function, light reactions) and pyrimidine nucleotide metabolism, likely reflecting accelerated senescence and energy deficits during extended storage. The study by Peng also revealed that with increasingly serious internal browning in Nane plum fruit, genes related to photosynthesis were downregulated, while genes related to senescence were upregulated [22]. This suppression correlated with the observed increase in core browning, as impaired metabolic processes may exacerbate cellular dysfunction. Furthermore, shared downregulation of hormone-responsive pathways (e.g., auxin, endogenous stimuli) across all stages underscores a systemic decline in stress adaptation capacity during storage.

The KEGG pathway analysis of ‘Yali’ pears under slow cooling revealed conserved stress response mechanisms, with genes upregulated across all maturity stages enriched in metabolic pathways, including secondary metabolite biosynthesis, terpenoid backbone, sesquiterpenoid, triterpenoid biosynthesis, and steroid biosynthesis. Notably, early- and mid-harvested pears shared enrichment in glycerophospholipid metabolism, plant hormone signal transduction, and MAPK signaling pathways, which likely contributed to delayed core browning via enhanced stress adaptation and membrane stability [23]. Mid-harvested pears uniquely exhibited enrichment in glycerolipid metabolism, nucleotide sugar biosynthesis, and DNA repair pathways, further supporting their resilience. Conversely, late-harvested pears demonstrated significant downregulation in pyruvate metabolism, GPI-anchor biosynthesis, and amino acid degradation pathways, implying that suppressed energy metabolism and membrane integrity maintenance exacerbated browning. Downregulated genes across all stages were linked to metabolic pathways, glyoxylate/dicarboxylate metabolism, and starch/sucrose metabolism, indicating a general metabolic slowdown. These findings collectively suggest that core browning in ‘Yali’ pears is influenced by both upregulated stress-adaptation pathways and downregulated metabolic processes, with mid-harvested pears exhibiting the most robust protective mechanisms.

Transcription factors (TFs) regulate gene expression by specifically binding to cis-acting elements, thereby constructing complex regulatory networks. These networks play a crucial role in plant responses to environmental stresses and the maintenance of developmental homeostasis [24]. Of particular interest was those associated with the RAV family transcription factors. The RAV family is a member of the B3 superfamily, which also encompasses the ARF, LAV, and REM families. The B3 superfamily is characterized by the presence of the B3 domain, a conserved region comprising approximately 110 amino acids [25]. RAV family transcription factors are implicated in the regulation of diverse plant physiological processes, including leaf senescence, floral development, organogenesis, and hormone signaling pathways [25]. The cis-element analysis in this study provided critical insights into the regulatory potential of the PbRAV transcription factor. The presence of growth-related motifs (e.g., CAT-box), abiotic stress-responsive elements (e.g., LTR, MBS), and hormone-associated sequences (e.g., ABRE, GARE-motif) in the promoter regions suggests that PbRAV integrates diverse signaling pathways to modulate stress adaptation and developmental processes. Notably, the abundance of hormone-responsive elements, particularly those linked to abscisic acid (ABRE) and gibberellin (P-box, TATC-box), implies that PbRAV may act as a nexus for hormonal regulation, potentially coordinating stress responses with growth dynamics during fruit storage. Zhao also found that the RAV family members mediate plant growth and the developmental process, and that these proteins are responsive to diverse hormonal/pathogenic bacterial stimuli [25]. The temporal expression profiles of *PbRAV* genes within ‘Yali’ pear cores further substantiate their functional significance. The biphasic upregulation of *PbRAV1* and *PbRAV2* in mid-harvested fruit, with peak expression at 90 d, is consistent with their putative role in attenuating core browning, as demonstrated by the reduced browning index observed in these samples. The significantly higher fold increase in *PbRAV2* expression compared to *PbRAV1* suggested a dominant regulatory role for *PbRAV2* in suppressing oxidative or enzymatic pathways driving browning. Conversely, the sustained downregulation of *PbRAV4* in late-harvested fruit correlates with accelerated browning, indicating that *PbRAV4* suppression may disrupt protective metabolic pathways, exacerbating oxidative damage.

While low-temperature storage effectively inhibits fruit senescence, this study employed a gradual cooling method to delay core browning in optimally mature ‘Yali’ pears. However, prolonged cold stress during storage can induce metabolic disturbances, leading to quality deterioration, including phenolic oxidation browning, cell wall structural alterations, and the loss of secondary metabolites [26], ultimately triggering core browning in the fruit. RAV transcription factors are implicated in modulating plant responses to both biotic and abiotic stressors [27]. However, the downstream regulatory network requires further investigation. The JASPAR-based prediction of PbRAV-binding motifs (5′-CAACA-3′ and 5′-CACCTG-3′) and the subsequent identification of 140,798 CAACA and 10,980 CACCTG sites within the P. bretschneideri genome underscore the expansive regulatory network orchestrated by PbRAV. KEGG enrichment analysis, which associates PbRAV with lipid metabolism and signal transduction pathways, supports its putative role in maintaining membrane integrity and the synthesis of stress-responsive metabolites. Previous studies also have reported that RAV transcription factors have a regulatory function in response to drought stress, salt stress, hormonal stress, and virus infection [25,27].

The lychee fruit-derived laccase (LAC), designated as LcADE/LAC, implicated in anthocyanin degradation, was identified as a key factor in pericarp browning [28]. Specifically, ADE/LAC-mediated flavonoid polymerization significantly contributes to the observed pericarp browning phenotype [29]. In this study, the specific binding of GST-PbRAV2 to the PbLAC15 promoter, as validated by EMSA, mechanistically links PbRAV2 to the regulation of laccase, a key enzyme involved in phenolic oxidation and browning. Competitive binding assays further confirm the specificity of this interaction, suggesting that it may participate in the core browning process of Yali pear fruit by regulating the upregulation of the LAC15 gene through the phenylpropanoid metabolic pathway.

## 5. Conclusions

This study investigates core browning mechanisms in postharvest ‘Yali’ pears under slow-cooling treatment. The analysis of three maturity stages revealed that slow cooling effectively reduced browning in early/mid-harvest fruit by preserving firmness and soluble solids. Transcriptomic profiling demonstrated conserved stress adaptation, as evidenced by the upregulation of specific genes across various maturity stages in ‘Yali’ pears subjected to slow-cooling treatment. The mid-harvest fruit uniquely exhibited the activation of phosphorus metabolic pathways and DNA repair mechanisms, thereby promoting cellular homeostasis and consequently delaying core browning. Conversely, the late-harvest fruit displayed suppressed photosynthetic activity and pyrimidine metabolism, leading to energy deficits and accelerated senescence. This maturity-dependent core browning mechanism was orchestrated by differential metabolic reprogramming: the mid-maturity pears maintained membrane integrity via enhanced lipid metabolism, whereas the late-maturity fruit showed downregulation of energy production pathways (e.g., pyruvate metabolism) and hormone-response pathways, collectively exacerbating browning progression. Key RAV transcription factors (e.g., PbRAV2) were linked to browning via regulation of downstream *LAC15* (laccase gene). EMSA confirmed PbRAV2 binds the PbLAC15 promoter, with dysregulation in overripe fruit impairing LAC15 suppression, accelerating browning. Future work will employ in vivo ChIP-qPCR on chilled pear tissue and transient expression assays in ‘Yali’ pear fruit to confirm regulatory functionality during browning progression. These findings provide mechanistic insights for optimizing slow-cooling strategies to mitigate postharvest losses.

## Data Availability

The original contributions presented in the study are included in the article/Supplementary Material, further inquiries can be directed to the corresponding author.

## Supplementary Materials

The following supporting information can be downloaded at: https://www.mdpi.com/article/10.3390/foods14122132/s1, Figure S1: The heat map for cluster analysis of RAV differentially expressed genes in the core of harvested ‘Yali’ pear; Figure S2: KEGG enrichment analysis of PbRAV downstream target gene (Note: Figure A: Contains CAACA binding site; Figure B: Contains CACCTG binding site). Table S1: Primer sequences used for qPCR validation; Table S2: PbRAV promoter sequence analysis.

## References

[1] Liu W., Zhang Y., Wang L., Ahmad B., Shi X., Ren Y., Liang C., Zhang X., Zhang Y., Du G. (2023). Integrated transcriptome and metabolome analysis unveiled the mechanisms of xenia effect and the role of different pollens on aroma formation in ‘Yali’ pear (*Pyrus bretschneideri* Rehd). Sci. Hortic..

[2] Zhang Y., Liu W., Shi X., Zhang Y., Du G. (2022). The characteristic of Yatu morphogenesis and the efficacy of exogenous hormones on the development of Yatu during fruit development in ‘Yali’ pear (*Pyrus bretschneideri* Rehd.). Plant Signal Behav..

[3] Dong Y., Zhi H. (2024). Applications of 1-methylcyclopropene concentrations and timing in relation to sunlight-related core browning in ‘Bartlett’ pears after extending regular-air storage. Postharvest Biol. Technol..

[4] Zhang H., Han Y., Liang L., Deng B. (2024). Rapid Cooling Delays the Occurring of Core Browning in Postharvest ‘Yali’ Pear at Advanced Maturity by Inhibiting Ethylene Metabolism. Foods.

[5] Li J., Yao T., Xu Y., Cai Q., Wang Y. (2022). Elevated CO_2_ exposure induces core browning in Yali pears by inhibiting the electron transport chain. Food Chem..

[6] Hao Y., Li X., Zhang C., Lei Z. (2023). Online Inspection of Browning in Yali Pears Using Visible-Near Infrared Spectroscopy and Interpretable Spectrogram-Based CNN Modeling. Biosensors.

[7] Li L., Zhang Y., Fan X., Wang J., Liang L., Yan S., Xiao L. (2020). Relationship between activated oxygen metabolism and browning of “Yali” pears during storage. J. Food Process Preserv..

[8] Yuan Q., Jiang Y., Yang Q., Li W., Gan G., Cai L., Li W., Qin C., Yu C., Wang Y. (2024). Mechanisms and control measures of low temperature storage-induced chilling injury to solanaceous vegetables and fruits. Front. Plant Sci..

[9] Liu L., Huang A., Wang B., Zhang H., Zheng Y., Wang L. (2024). Melatonin mobilizes the metabolism of sugars, ascorbic acid and amino acids to cope with chilling injury in postharvest pear fruit. Sci. Hortic..

[10] Jia L., Li L., Luo W., Zhang X., Zhu L., Qian M., Gu P., Xie Y., Yang B., Qiao X. (2024). PbrWRKY42-PbrSOT13 module regulated sorbitol accumulation in the developing ‘Yali’ fruit after three-layer-paper bagging treatment. Sci. Hortic..

[11] Zhao J., Zou Q., Bao T., Kong M., Gu T., Jiang L., Wang T., Xu T., Wang N., Zhang Z. (2024). Transcription factor MdbZIP44 targets the promoter of MdPPO2 to regulate browning in Malus domestica Borkh. Plant Physiol. Biochem..

[12] Sun H.-J., Luo M.-L., Zhou X., Zhou Q., Sun Y.-Y., Ge W.-Y., Yao M.-M., Ji S.-J. (2020). PuMYB21/PuMYB54 coordinate to activate PuPLDβ1 transcription during peel browning of cold-stored “Nanguo” pears. Hortic. Res..

[13] Duan W., Yang C., Cao X., Zhang C., Liu H., Chen K., Li X., Zhang B. (2022). Transcriptome and DNA methylome analysis reveal new insights into methyl jasmonate-alleviated chilling injury of peach fruit after cold storage. Postharvest Biol. Technol..

[14] Bielsa F.J., Grimplet J., Irisarri P., Miranda C., Errea P., Pina A. (2025). Comparative enzymatic browning transcriptome analysis of three apple cultivars unravels a conserved regulatory network related to stress responses. BMC Plant Biol..

[15] Mu H., Chen J., Huang W., Huang G., Deng M., Hong S., Ai P., Gao C., Zhou H. (2024). OmicShare tools: A zero-code interactive online platform for biological data analysis and visualization. Imeta.

[16] Fan Z.-Q., Ba L.-J., Shan W., Xiao Y.-Y., Lu W.-J., Kuang J.-F., Chen J.-Y. (2018). A banana R2R3-MYB transcription factor MaMYB3 is involved in fruit ripening through modulation of starch degradation by repressing starch degradation-related genes and MabHLH6. Plant J..

[17] Tan X.-L., Fan Z.-Q., Shan W., Yin X.-R., Kuang J.-F., Lu W.-J., Chen J.-Y. (2018). Association of BrERF72 with methyl jasmonate-induced leaf senescence of Chinese flowering cabbage through activating JA biosynthesis-related genes. Hortic. Res..

[18] Yan S., Li L., He L., Liang L., Li X. (2013). Maturity and cooling rate affects browning, polyphenol oxidase activity and gene expression of ‘Yali’ pears during storage. Postharvest Biol. Technol..

[19] Kaur K., Dhillon W.S. (2016). Effect of harvesting date and packaging materials on core browning and phenolic contents of pear cv. Punjab Beauty during storage. Ind. J. Hort..

[20] Lwin H.P., Torres C.A., Rudell D.R., Lee J. (2023). Chilling-related browning of ‘Wonhwang’ pear cortex is associated with the alteration of minerals and metabolism. Sci. Hortic..

[21] Gao C., Zhang Y., Li H., Gao Q., Cheng Y., Ogunyemi S.O., Guan J. (2022). Fruit bagging reduces the postharvest decay and alters the diversity of fruit surface fungal community in ‘Yali’ pear. BMC Microbiol..

[22] Peng C., Deng L., Tan H., Meng W., Luo J., Zhang Z., Chen H., Qiu J., Chang X., Lu Y. (2024). Transcriptome and metabolome analysis of preharvest internal browning in Nane plum fruit caused by high temperature. Hortic. Plant J..

[23] Liang H., Zhu Y., Li Z., Jiang Y., Duan X., Jiang G. (2025). Phytosulfokine treatment delays browning of litchi pericarps during storage at room temperature. Postharvest Biol. Technol..

[24] Liu W., Liang X., Cai W., Wang H., Liu X., Cheng L., Song P., Luo G., Han D. (2022). Isolation and Functional Analysis of VvWRKY28, a Vitis vinifera WRKY Transcription Factor Gene, with Functions in Tolerance to Cold and Salt Stress in Transgenic Arabidopsis thaliana. Int. J. Mol. Sci..

[25] Zhao S.-P., Xu Z.-S., Zheng W.-J., Zhao W., Wang Y.-X., Yu T.-F., Chen M., Zhou Y.-B., Min D.-H., Ma Y.-Z. (2017). Genome-Wide Analysis of the RAV Family in Soybean and Functional Identification of GmRAV-03 Involvement in Salt and Drought Stresses and Exogenous ABA Treatment. Front. Plant Sci..

[26] Zhu X., Luo J., Li Q., Li J., Liu T., Wang R., Chen W., Li X. (2018). Low temperature storage reduces aroma-related volatiles production during shelf-life of banana fruit mainly by regulating key genes involved in volatile biosynthetic pathways. Postharvest Biol. Technol..

[27] Chen C., Li Y., Zhang H., Ma Q., Wei Z., Chen J., Sun Z. (2021). Genome-Wide Analysis of the RAV Transcription Factor Genes in Rice Reveals Their Response Patterns to Hormones and Virus Infection. Viruses.

[28] Zhang X., Fang F., He Q., Zhang X., Shi N., Song J., Zhang Z., Pang X. (2018). Enzymatic characterization of a laccase from lychee pericarp in relation to browning reveals the mechanisms for fruit color protection. J. Food Process Preserv..

[29] Wei J., Zhang X., Zhong R., Liu B., Zhang X., Fang F., Zhang Z., Pang X. (2021). Laccase-Mediated Flavonoid Polymerization Leads to the Pericarp Browning of Litchi Fruit. J. Agric. Food Chem..

