# Supplementary material for: Mechanism of Core Browning in Different Maturity Stages of ‘Yali’ Pears During Slow-Cooling Storage and PbRAV-Mediated Regulation
Source: Foods. 2025 Jun 18;14(12):2132. doi: 10.3390/foods14122132 (PMC12191683; doi:10.3390/foods14122132)
Supplement: Supplementary file 1 [file foods-14-02132-s001.zip › foods-3660924-supplementary.pdf]

Supplementary figures and tables

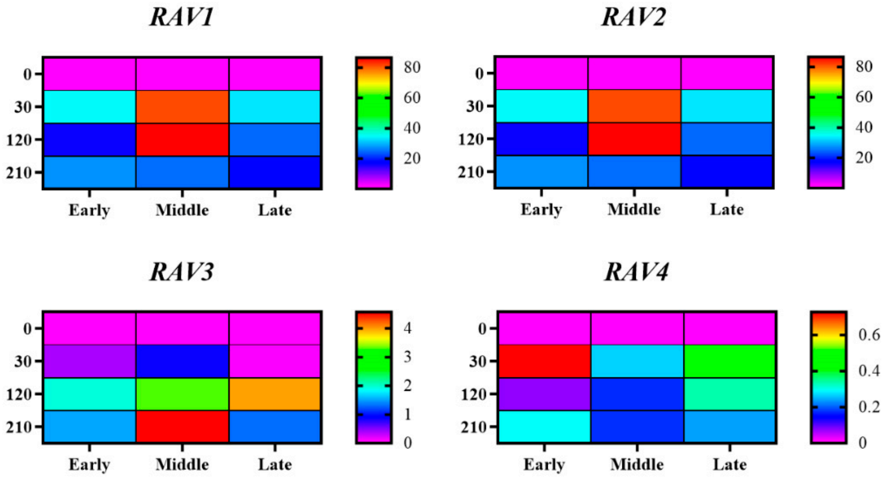

Figure S1. The heat map for cluster analysis of RAV differentially expressed genes in the core of harvested 'Yali' pear.

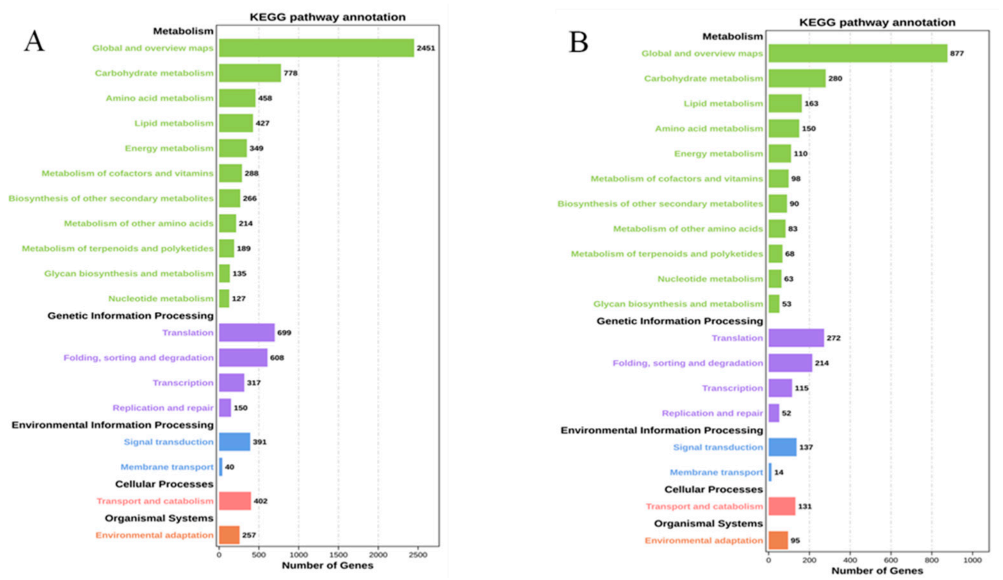

Figure S2. KEGG enrichment analysis of PbRAV downstream target gene (Note: Figure A: Contains CAACA binding site; Figure B: Contains CACCTG binding site).

Table S1. Primer sequences used for qPCR validation

Gene name	Gene ID	Primer Sequence 5′→3′	Product length
PbActin	*GU830958.1*	F:TGGTATGGGTCAGAAGG<br>R:CAGGAGCAACACGAAGT	161
PbRAV1	*XM_048572084.1*	F： GGGGTGCCCAGATTTA<br>R： TCGACGATTTCGGACTT	226
PbRAV2	*XM_009357987.3*	F： TAAAGCCCTCCTCCG<br>R： CGACTGTTGTCCATCCC	220

PbRAV3	*XM_009351004.2*	F：GCTGCTAAGACCTACGA<br>R：GACCACCATCACGAACT	229
PbRAV4	*XM_009358762.3*	F：TACGACATTGCCTCGCTAA<br>R：AACACTTCATACGCCTTCC	250

Table S2. PbRAV promoter sequence analysis.

Elementname	Coresequence	Function
ABRE	ACGTG	Involved in abscisic acid response.
GARE-motif	TCTGTTG	Involved in gibberellin response.
P-box	CCTTTTG	Involved in gibberellin response.
TATC-box	TATCCCA	Involved in gibberellin response.
TCA-element	TCAGAAGAGG	Involved in salicylic acid response.
TGACG-motif	TGACG	Involved in MeJA response.
CGTCA-motif	CGTCA	Involved in MeJA response.
AuxRR-core	GGTCCAT	Involved in auxin response.
CAT-box	GCCACT	Involved in meristem expression-related regulation.
ARE	AAACCA	Involved in anaerobic induction regulation.
TC-richrepeats	GTTTTCTTAC	Involved in defense and stress responses.
LTR	CCGAAA	Involved in low-temperature response.
MBS	CAACTG	Involved in drought induction (MYB binding site).
CCAAT-box	CAACGG	MYBHv1 binding site.
TATA-box	TATAAAT	Core promoter region near transcription start site -30.
GC-motif	CCCCCG	Involved in hypoxia-specific induction.
CAAT-box	CAAAT/CCAAT	Common elements in promoter and enhancer regions.
G-Box	CACGTT/CACGTC	Involved in light response.
MBIS	aaaAaaC(G/C) GTTA	Involved in flavonoid biosynthesis gene regulation (MYB binding site).
